# Use of health economic evaluation in the implementation and improvement science fields—a systematic literature review

**DOI:** 10.1186/s13012-019-0901-7

**Published:** 2019-07-15

**Authors:** Sarah Louise Elin Roberts, Andy Healey, Nick Sevdalis

**Affiliations:** 1 King’s Health Economics, Health Service and Population Research Department, Institute of Psychiatry, Psychology and Neuroscience, King’s College London, David Goldberg Centre, De Crespigny Park, London, SE5 8AF UK; 2 Centre for Implementation Science, King’s College London, London, UK

## Abstract

**Background:**

Economic evaluation can inform whether strategies designed to improve the quality of health care delivery and the uptake of evidence-based practices represent a cost-effective use of limited resources. We report a systematic review and critical appraisal of the application of health economic methods in improvement/implementation research.

**Method:**

A systematic literature search identified 1668 papers across the Agris, Embase, Global Health, HMIC, PsycINFO, Social Policy and Practice, MEDLINE and EconLit databases between 2004 and 2016. Abstracts were screened in Rayyan database, and key data extracted into Microsoft Excel. Evidence was critically appraised using the Quality of Health Economic Studies (QHES) framework.

**Results:**

Thirty studies were included—all health economic studies that included implementation or improvement as a part of the evaluation. Studies were conducted mostly in Europe (62%) or North America (23%) and were largely hospital-based (70%). The field was split between improvement (*N* = 16) and implementation (*N* = 14) studies. The most common intervention evaluated (43%) was staffing reconfiguration, specifically changing from physician-led to nurse-led care delivery. Most studies (*N* = 19) were ex-post economic evaluations carried out empirically—of those, 17 were cost effectiveness analyses. We found four cost utility analyses that used economic modelling rather than empirical methods. Two cost-consequence analyses were also found. Specific implementation costs considered included costs associated with staff training in new care delivery pathways, the impacts of new processes on patient and carer costs and the costs of developing new care processes/pathways. Over half (55%) of the included studies were rated ‘good’ on QHES. Study quality was boosted through inclusion of appropriate comparators and reporting of incremental analysis (where relevant); and diminished through use of post-hoc subgroup analysis, limited reporting of the handling of uncertainty and justification for choice of discount rates.

**Conclusions:**

The quantity of published economic evaluations applied to the field of improvement and implementation research remains modest; however, quality is overall good. Implementation and improvement scientists should work closely with health economists to consider costs associated with improvement interventions and their associated implementation strategies. We offer a set of concrete recommendations to facilitate this endeavour.

## Background

Both improving health care and implementation of evidence-based practices are receiving increasing attention within the wider applied health research field. A recent editorial in *Implementation Science* [1] discussed the importance implementation science places on the robustness and validity of health economic evaluations and the benefits gained by properly evaluating both implementation and improvement interventions. We define *improvement* science as the scientific approach to achieving better patient experience and outcomes through changing provider behaviour and organisation, using systematic change methods and strategies [2]. We define *implementation* science as the scientific study of methods to promote the uptake of research findings into routine health care practice or policy [2].

This paper presents a review of the application of economic evaluation to evaluative studies of service improvement initiatives and interventions focused on facilitating the implementation of evidence into practice. The aim of economic evaluation is to present evidence on the costs and consequences (in terms of patient outcomes) of quality improvement strategies and methods for increasing the uptake of evidence-based practices compared to the ‘status quo’. In doing so, it informs whether specific initiatives are (or have been) a worthwhile (or ‘cost-effective’) use of the limited resources of health systems.

Depending on the service and population context, the methods used in economic evaluations can vary depending on the perspective taken. This can range from a narrow assessment of patient outcomes alongside immediate health care provider cost impacts through to the quantification of costs and consequences affecting other (non-health related) sectors, organisations and wider society. In health programme evaluation, economic evaluations are most frequently carried out ‘ex-post’ or ‘after the fact’, using empirical methods applied to cost and outcome data extracted from trials or other research designs used to evaluate initiatives being tested in specific populations and settings. Economic evaluations can also be applied ‘ex-ante’—to inform option appraisal and pre-implementation decision making using available evidence and modelling to simulate the costs and outcomes of alternatives, e.g. in relation to population scale up or geographical spread of strategies and methods for improvement and evidence uptake.

While economic evaluation has become an integral part of health technology assessment, its application within improvement and implementation evaluative research remains relatively limited [1]. In two earlier reviews (Hoomans et al. in 2007 [3] and earlier Grimshaw et al. in 2004 [4]), the use of economic methods in evaluating the implementation of evidence-based guidelines was examined, and the authors found evidence of limited quality and scope for understanding the cost-effectiveness of implementation strategies. It is now over a decade since these reviews were published, hence a fresh evidence review, synthesis and appraisal is required.

The aim of this study was to examine what advances have been made in the use of economic analysis within implementation and improvement science research, specifically in relation to the quantity and quality of published economic evidence in this field; and to what extent economic evaluations have considered implementation and improvement as part of a holistic approach to evaluating interventions or programmes within the applied health arena.

## Methods

### Search strategy

A systematic review methodology was undertaken. A search strategy was developed to capture evidence published after 2003 (the date of most recent evidence review) and the last searches were performed on 16th March 2016. The searches were performed on the following databases: Agris, Embase, Global Health, HMIC, PsycINFO, Social Policy and Practice, MEDLINE and EconLit. These databases were chosen to attempt to capture the widest range of health improvement, social scientific and health economic studies.

The search strategy (Table 1) was designed to capture studies that had a quantitative economic element (i.e. costs and outcomes based on randomised trial data, observational study data or synthesis of the wider empirical evidence base to support economic modelling). The search was conducted to be inclusive of studies whereby behavioural interventions for quality improvement and implementation of evidence into practice were evaluated as well as initiatives around re-design or adjustment to care pathways or reconfiguration of staffing inputs for the purpose of quality improvement.

**Table 1:** Search strategy for the systematic review

SEARCH 1: economic or evaluation or cost effect* or “cost saving” AND improv* or ‘behaviour change’ or ‘willingness to change’ or accept* or ‘roll out’ or change or adhere* AND ‘clinical guideline*’ or ‘education outreach’ or evidence or ‘evidence based’ or ‘quality improv*’ or ‘service improv* or local impl*’ AND clinical or doctor or nurse or ‘allied health professionals’ or clinician or pathway or ‘decision make*’ or ‘local govern*’ or ‘clinical commiss*’ or ‘commissioners’ o Including limited related terms SEARCH 2: economic or evaluation or ‘cost effect*’ or ‘cost saving’ AND improv* or ‘behavior change’ or ‘willingness to change’ or accept* or ‘roll out’ or change or adhere* AND ‘clinical guideline*’ or ‘education outreach’ or evidence or ‘evidence based’ or ‘quality improv*’ or ‘service improv*’ or ‘local impl*’ AND clinical or doctor or nurse or ‘allied health professionals’ or clinician or pathway or ‘decision make*’ or ‘local govern*’ or ‘clinical commiss*’ or ‘commissioners’ o Including related terms SEARCH 3: search 1 without related terms SEARCH 4: search 2 without related terms	

We searched across a wide range of clinical settings, including primary, secondary and tertiary care and public health.

### Screening

The completed search results were downloaded into Endnote X6 for citation management and deduplication. Screening was done in Rayyan, a web-based literature screening program [6]. Rayyan allows for easy abstract and full text screening of studies, custom inclusion and exclusion criteria, as well as custom tags or labels that can be added to each entry. Studies were initially screened using the inclusion/exclusion criteria outlined in the next section, on title and abstract only (by SLER); studies that were borderline for inclusion were more thoroughly screened by examining their full text. The reference lists of the studies were checked for any related studies that were not picked up by the search.

### Inclusion and exclusion criteria

Studies were included if they:

- Were published in the English language
- Reported on a completed study
  - Study protocols, methodological papers or conference abstracts were excluded (after additional searches had been performed to ensure that full papers had not been subsequently published).
- Were published after 2003 and before 16th March 2016
- Were conducted in public health, primary, secondary or tertiary care

Further, studies were included if they covered aspects of:

- Implementation
- Quality/service improvement
- Health or clinical service delivery
- Staff behaviour change
- Patient behaviour change

And they also:

- Had patient focused outcomes or outcomes as overall service improvement that would improve patient outcomes or care, expressed as quantifiable outcomes
- Had economic elements, expressed as quantifiable outcomes
- Reported one of the following health economic methodologies:
  - Cost effectiveness analysis
  - Cost-utility analysis
  - Cost-benefit analysis
  - Cost-consequence analysis
  - Burden of disease

The following study designs were included:

- Randomised controlled trials
- Hybrid effectiveness-implementation trials
- Comparative controlled trials without random assignment
- Before and after studies
- Systematic reviews
- Time series study design

Studies or papers that did not fall within the above criteria were excluded. No geographical exclusions were applied. Cost-only studies were not included as the aim of this review was to establish the extent that both costs and benefits were being considered as part of a holistic approach to evaluation of implementation and improvement interventions.

To mitigate for potential selection bias after screening, keyword searching was done in Rayyan for the main keywords within the excluded categories (primarily, those that were deemed to be topic-relevant but not containing economic methods). These were then re-screened by the first author. Studies that included only minimal discussion of costs or costing with no evidence of application of appropriate, standard costing methods (as per the criteria above) were excluded.

### Data extraction

Screened studies were downloaded from Rayyan and transferred into a template developed in Microsoft Excel 2016 for detailed data extraction. During screening, each included study was tagged in Rayyan with the reasons for inclusion, type of economic evaluation (see Table 2), which economic modelling method used (if applicable), whether improvement or implementation study, the health condition covered, the focus of the reported intervention and health care setting. These were cross-checked for accuracy during the data extraction stage. The next stage of the extraction added the country of the study, perspective of the study (healthcare only or ‘societal’), and more detailed information about the economic methods. The latter included whether the evaluation included appropriate comparators (e.g. status quo/the standard care practice), patient outcome measures used, whether costs and outcomes were analysed and reported in the form of incremental cost-effectiveness ratios (ICERs) for cost-effectiveness or cost-utility analyses, how uncertainty was handled and what conclusions were made regarding the cost-effectiveness of the interventions under evaluation.

**Table 2:** Types of economic analysis included in the review

Cost-consequences analysis (CCA): compares costs and multiple measures of patient outcome of alternatives under evaluation. Cost-effectiveness analysis (CEA): compares costs and outcomes of alternatives using a single primary measure of patient outcome (e.g. life-years gained; cases of disease avoided; improvements in clinical functioning; improvements in quality of care experience). Cost-utility analysis (CUA): compares costs and outcomes of alternatives with outcomes measured as quality-adjusted life years (QALYs) gained. Cost-benefit analysis (CBA): compares costs and outcomes of alternatives, with patient outcomes valued monetarily. Cost-analysis (CA): costs implications only of relevant alternatives evaluated with no consideration of impact on quality of care and patient outcomes (not strictly a full economic evaluation).	

### Quality appraisal

Each paper’s methodological quality was assessed using the Quality of Health Economic Studies (QHES) standardised framework [4]. The QHES instrument was designed to more easily tell the difference between high-quality and low-quality studies [5]. Each study was scored out of 100 based on 16 criteria, with points allocated for full and partial assessments against each item (see Appendix in Table 7 for the framework and scoring system). As per standard practice using this framework, the studies were deemed to be of good quality if they attained a score of 75/100 or higher [5].

## Results

### Studies included

**Fig. 1:**
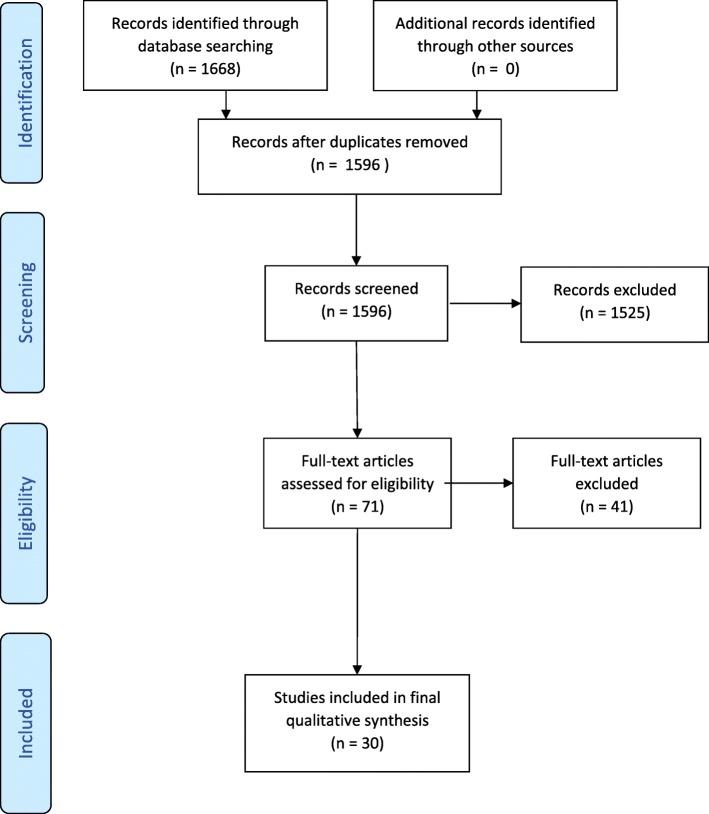
Consort diagram

Figure 1 shows the flow of studies through the screening stages of the systematic review.

In total, the initial search strategy identified 1668 articles, of which 1566 were excluded, 1525 during the initial screen and 41 following full text screening. Reasons for exclusion were as follows: the study did not include implementation or quality improvement research aspects (575); it did not include economic aspects (447); was not within a health care/public health setting (437); it was in a language other than English (22); it was incomplete (19); or it was not a full refereed publication (e.g. conference abstracts, doctoral theses) (37).

Thirty studies were included in the final evidence review and synthesis.

### Descriptive analysis of the evidence base

Table 3 provides a descriptive overview of the evidence base reported in the 30 reviewed studies. Seventeen of the studies (62%) were European-based (mostly from the UK—12 studies), six studies (23%) were based in either the USA or Canada, four from Australia and one each from Ethiopia, a subset of African countries (Uganda, Kenya and South Africa) and Malaysia. In terms of health care settings, 21 studies were hospital-based, approximately half in inpatient wards and departments, including cardiology, oncology, rheumatology, gastroenterology, geriatrics, endocrinology, orthopaedics and respiratory medicine, or specifically concerning ward management or discharge protocols.

**Table 3:** Summary of included studies and quality appraisal—panel a improvement studies; panel b implementation studies

Author	Year	Country	Improvement or implementation focus	Care setting	Improvement intervention focus	Sample size	Main study outcomes	Type of economic analysis	Quality appraisal score for economic modelling (out of 100)
a: Improvement studies									
Afzali et al	2013	Australia	Improvement	Endocrinology	Staff mix reformulation (nurse-led)	3642	EQ-5D (EuroQol 5 dimension scale)	CEA	100
Albers-Heitner et al	2010	Netherlands	Improvement	Primary Care	Staff mix reformulation (nurse-led)	384	EQ-5D	CEA	74
Bauer	2010	USA	Improvement	N/A	Staff mix reformulation (nurse-led)	160	Resource use	CEA	N/A
Dawes et al	2007	UK	Improvement	Gynaecology	Staff mix reformulation (nurse-led)	111	SF-36 (36-Item Short Form Health Survey), Length of Stay	CCA	66.5
Faulkener et al	2003	UK	Improvement	Primary Care	Improved referral	N/A	Review	Review	N/A
Furze et al	2011	UK	Improvement	Cardiology	Staff mix reformulation (peer support)	142	EQ-5D	CUA	84.5
Hernandez et al	2014	UK	Improvement	Intensive Care	Staff mix reformulation (nurse-led)	286	EQ-5D	CUA	90.5
Karnon et al	2016	Australia	Improvement	Cardiology	Service Reconfiguration (funding sources)	603	N/A	CCA	44.5
Kilpatrick et al	2014	Canada	Improvement	Hospital General Medicine	Staff mix reformulation (nurse-led)	2147	Review	CEA	N/A
Latour et al	2007	Netherlands	Improvement	Hospital wards	Staff mix reformulation (nurse-led)	208	SF-36, HADS (Hospital Anxiety and Depression Scale)	CEA	81.5
Mdege et al	2012	Sub-Saharan Africa	Improvement	HIV	Staff mix reformulation (multiple scenarios)	19,767	N/A	Review	N/A
Tappenden et al	2012	UK	Improvement	Geriatrics	Staff mix reformulation (nurse-led)	N/A	Review	CEA	N/A
Walsh et al	2005	UK	Improvement	General Medicine	Staff mix reformulation (nurse-led)	238	Bed days	CA	65
Williams et al	2006	UK	Improvement	Gastroenterology	Staff mix reformulation (nurse-led)	1500	EQ-5D	CEA	94
Williams et al	2005	UK	Improvement	Urology	Staff mix reformulation (nurse-led)	3746	EQ-5D	CEA	51
Yarbrough et al	2015	USA	Improvement	General Medicine	New pathway	677	Resource use	CEA	N/A
b: Implementation studies									
Author	Year	Country	Improvement or implementation focus	Care setting	Implementation intervention focus	Main study outcomes	Type of economic evaluation	Sample size	Quality appraisal score for economic modelling (out of 100)
Brunenberg et al	2005	Netherlands	Implementation	Orthopaedics	Pathway implementation	EQ-5D, Length of stay	CEA	160	71
Burr et al	2007	UK	Implementation	Ophthalmology	Screening programme implementation	EQ-5D	CUA	207–32,918	89.5
Burr et al	2012	UK	Implementation	Ophthalmology	Surveillance programme implementation	EQ-5D, Willingness to pay	CUA	800	92.5
Judd et al	2014	USA	Implementation	Hospital wards	Early intervention implementation	Length of Stay	CA	181	37
Kifle et al	2010	Ethiopia	Implementation	All hospital specialities	Referral system implementation	Resource use	CEA	532	N/A
Maloney et al	2012	Australia	Implementation	Physiotherapy	Health professional education	Costs only	CEA	85	94.5
Mortimer et al	2013	Australia	Implementation	General Practice	Implementation methods (active vs guideline dissemination)	EQ-5D, X rays avoided	CEA	112	81.5
Purshouse et al	2013	UK	Implementation	Public Health	Screening programme implementation	EQ-5D	CEA	N/A	82
Rachev	2015	USA	Implementation	Public Health	General methods of health service transformation	Resource use	CEA	N/A	N/A
Robertson et al	2011	UK	Implementation	Oncology	Surveillance programme implementation	EQ-5D	CUA	N/A	94
Tappenden et al	2013	UK	Implementation	Oncology	Resource allocation decision making	EQ-5D	CUA	N/A	84
Umscheid et al	2010	Canada	Implementation	N/A	Comparative effectiveness centre	None	Review	N/A	N/A
Vestergaard et al	2015	Denmark	Implementation	Cardiology	Guideline adherence vs observed treatment	EQ-5D	CEA	N/A	57.5
Yee and Shafie	2013	Malaysia	Implementation	Respiratory	Asthma management implementation	EQ-5D	Review	N/A	N/A

Sixteen of the included studies were identified as ‘improvement’ studies (see Table 3, panel 1a) and 14 were identified as ‘implementation’ studies (see Table 3, panel 1b). The definitions from Batalden and Davidoff (2007) that are cited in the introduction were used to stratify the studies. The most common focus of the reviewed improvement studies was staff reconfigurations within a clinical area from medical to nursing staff; for implementation studies, the most common focus was on implementation strategies of new care pathways or novel services.

Table 4 summarises the types of intervention evaluated. The most common intervention type, evaluated in 13 (43%) of the included studies, was staffing reconfiguration for service quality improvement, specifically changing from physician-led to nurse-led delivery of interventions to patients. More broadly, interventions involving general service reorganisation or changes to existing systems of care were the primary focus in ten (33%) of studies reviewed.

**Table 4:** Focus of improvement/implementation intervention included in the reviewed evidence

Improvement or implementation interventions across studies (*N* of studies and %)		
Staffing reconfiguration	13	43%
Pathway implementation	4	14%
Review of practice	3	10%
Improvement in patient screening	3	10%
Service reconfiguration	2	7%
Improvement in follow up procedures	2	7%
Monitoring activity	1	3%
Guideline adherence	1	3%
Education	1	3%

Nineteen studies were ex-post economic evaluations of which 17 were CEAs with one CUA [7, 12, 14, 15, 17] [18–30, 33]. All these evaluations compared a new intervention against current practice. There were also four further CUAs that used economic modelling rather than empirical methods [8–10, 34], and two cost-consequence analyses [16, 35]. Three of the included studies were literature reviews [11, 13, 36].

Specific implementation costs, such as those associated with training staff in new care delivery pathways, the impacts of new processes on patient and carer costs and the costs of developing the new processes were considered by six of the reviewed studies. Scenario analysis for rollout or scaling up was included in three of the studies, and potential funding sources were considered by one study.

### Quality appraisal

**Fig. 2:**
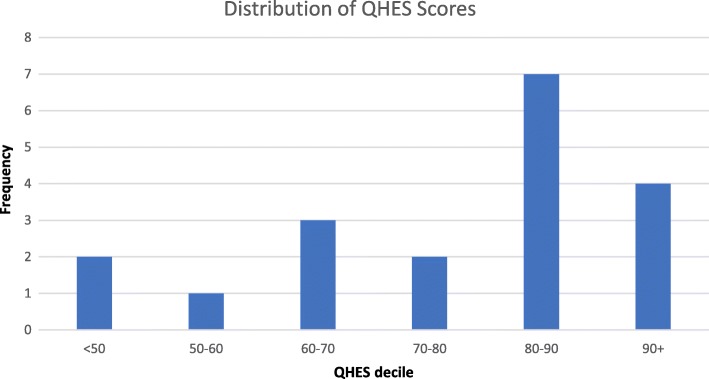
Quality appraisal of economic evidence—distribution of QHES instrument scores

Twenty-two of the papers were included in the QHES economic quality appraisal: as the quality scale is designed to evaluate cost-minimisation, cost-effectiveness and cost-utility studies [5], the literature reviews, meta-analyses or commentaries were excluded for this component. Of the excluded papers, four were systematic reviews and four were papers that did not report on specific studies. The QHES instrument contains 16 dimensions and an outline of the dimensions, the average score and the percentage of the papers reaching the perfect score for each dimension can be found in Table 5. While most of the papers in this study reached the threshold of being ‘good’ studies, the scores are gained mostly in the same areas in each paper. The average quality score was 76 out of a possible 100 (Fig. 2). Thirteen of the studies (62%) attained a ‘good’ score of over 75. Only one study [33] obtained a ‘perfect’ score of 100 points. Improvement studies performed overall better than implementation studies on the QHES.

**Table 5:** Summary of implementation costs and scenarios included

Study	Costs considered	Scenarios considered	Conclusion: intervention cost-effective?
Furze et al. 2011	Training costs	None	Yes
Judd et al. 2014	None	Scaling scenarios	Yes
Kifle et al. 2010	Indirect costs of patients and carers; project costs; impacts on staff	None	Yes
Maloney et al. 2012	Training and set up costs	Roll out scenarios	Yes
Mdege et al. 2012	Training costs	Roll out scenarios	Yes
Mortimer et al. 2013	Development costs; amortisation; delivery costs; roll out costs	Roll out scenarios	No
Purshouse et al. 2013	None	Roll out scenarios	Yes, although sensitive to rollout costs
Rachev 2015	Outlining of costs	None	Inconclusive
Tappenden et al. 2013	None	Funding scenarios	N/A

The best performing QHES dimensions were the methodological dimensions. Incremental analysis with a relevant comparator (dimension 6) was used in all but one study, and in 81% of studies the data sources for the analysis were from randomised controlled trials, the highest scoring type of evidence in the QHES instrument (Table 6). The costing element, covered by dimension 9, performed poorly overall. While three quarters of studies gave details of what methodology was used to quantify service inputs (such as use of self-report service use schedules) and the sources and methods used for estimating unit costs, only two gave justification for why they chose that method. By comparison, there was justification for the use of effectiveness measures and study outcomes given in two-thirds of studies.

**Table 6:** Summary of QHES instrument dimension scores

QHES dimension	Average score	Highest possible score	Percentage achieving highest possible score
Was the study objective presented in a clear, specific and measurable manner?	6.0	7	65%
Were the perspective of the analysis (societal, thirdparty, payer, etc.) and reasons for its selection stated?	2.4	4	28%
Were variable estimates used in the analysis from the best available source (i.e. randomised control trial—best, expert opinion—worst)?	7.4	8	83%
If estimates came from a subgroup analysis, were the groups prespecified at the beginning of the study?	0.4	1	33%
Was uncertainty handled by (1) statistical analysis to address random events, (2) sensitivity analysis to cover a range of assumptions?	5.8	9	33%
Was incremental analysis performed between alternatives for resources and costs?	5.4	6	94%
Was the methodology for data extraction (including the value of health states and other benefits) stated?	4.0	5	78%
Did the analytic horizon allow time for all relevant and important outcomes? Were benefits and costs that went beyond 1 year discounted (3% to 5%) and justification given for the discount rate?	4.7	7	39%
Was the measurement of costs appropriate and the methodology for the estimation of quantities and unit costs clearly described?	3.9	8	0%
Were the primary outcome measure(s) for the economic evaluation clearly stated and did they include the major short-term? Was justification given for the measures/scales used?	4.7	6	67%
Were the health outcomes measures/scales valid and reliable? If previously tested valid and reliable measures were not available, was justification given for the measures/scales used?	5.0	7	72%
Were the economic model (including structure), study methods and analysis, and the components of the numerator and denominator displayed in a clear, transparent manner?	6.7	8	83%
Were the choice of economic model, main assumptions, and limitations of the study stated and justified?	5.6	7	78%
Did the author(s) explicitly discuss direction and magnitude of potential biases?	3.9	6	56%
Were the conclusions/recommendations of the study justified and based on the study results?	8.0	8	100%
Was there a statement disclosing the source of funding for the study?	2.4	3	78%

Discount rates were correctly applied and stated when adjusting for timing of costs and benefits in all cases where measured costs and outcomes extended beyond 1 year.

A little over a quarter of the included studies declared the perspective of their analysis and gave a justification for the perspective used. Only a third gave details of how parameter uncertainty was addressed in relation to the study conclusions. Justification for chosen discount rates was not provided in around half the studies that used them. Where subgroup analysis was carried out, this was done post-hoc rather than being pre-planned with a clear a priori justification for the use of the chosen subgroups.

## Discussion

### Reflections on the evidence

The aim of this review was to critically evaluate the application of economic analysis within implementation and service improvement evaluative research in recent years. The results of evaluating the 30 included papers paint a picture of an area of research that is still developing. The reviewed studies were generally of good quality. However, we found that there were aspects of improvement and implementation that were not adequately covered in many studies. These reflect particularly project costs relating to managerial and clinical time allocated to preparatory work and training and education as well as ongoing costs linked to monitoring care quality and outcomes—all of which are known strategies for successful implementation [37]. Only six out of 30 studies included an explicit assessment of these type of ‘hidden’ costs of improvement and implementation strategies. This risks underestimating the cost impacts of change and could represent a missed opportunity to develop evidence about the likely comparative magnitude and importance of fixed and recurrent costs that are integral to the scale up and spread of improvement- and implementation-focussed initiatives.

A further reflection: many of the economic studies picked up in our review were linked to wider studies built around more traditional evaluative research designs, specifically randomised controlled trials. There was no evidence that economic methods have as yet been integrated into more advanced evaluative designs within the fields of improvement and implementation design, particularly ‘hybrid’ designs [38, 39] that aim to jointly test clinical effectiveness of the evaluated health intervention on patient outcomes and, simultaneously, effectiveness of implementation strategies in embedding the clinical intervention within an organisation or service. This may reflect the fact that hybrid designs are a more recent methodological development, which requires further integration into traditional health care evaluations.

Furthermore, and in relation to the wider role of health economic evaluations within the improvement and implementation science arena, we found that all of the studies included in our review were empirical and ex-post in nature. The studies evaluated costs and outcomes retrospectively using data over a period of time following the introduction of a specific improvement or implementation initiative. This is certainly valuable information for decision makers in making decisions about already applied interventions and in building up an economic evidence base around these interventions. However, it also suggests that economic analysis, and particularly economic modelling, currently at least appears to have a less important role in informing decisions over which options to pursue at earlier stages of implementing change, and in the appraisal of spread and scale up within wider populations. Such earlier phase economic analyses were simply not found in our review. We reflect that either this type of economic analysis is not happening—hence there is a significant gap in the application of economic considerations in improvement and implementation policy decisions; or that such analyses may indeed be undertaken but being less likely to be reported in academic publications and thus under-represented in our review. We cannot rule out either possibility based on this review. Our collective experience suggests that more nuanced economic analyses than simply consideration of ‘costs’ should be carried out in early phases of implementation and improvement programme planning; prospective economic modelling offers a way forward for health care improvers and policy makers planning scale up of evidence interventions.

### Quality of the evidence

Comparison between economic studies identified in a previous review carried out by Hoomans et al. (covering the immediately preceding period 1998 to 2004) with those identified in this review (2004 to 2016) shows evidence of a general improvement in quality over the past two decades, with the caveat that the two reviews used different quality appraisal frameworks. For example, only 42% of studies reviewed by Hoomans et al. included evaluation of costs and outcomes against ‘standard practice/status quo’ comparators, compared to 95% of studies in our review. Likewise, costing methodology was only deemed adequate in 11% of cases included in the Hoomans et al. review, compared to 76% of the studies in this review. Justification for the outcome measures used was not reported in any of the studies included in Hoomans et al. but reported in 68% of studies included here. This is a welcome improvement of applied economics within health care implementation and improvement research. We attribute it at least partly to improvements in reporting economic analyses over time, which would appear to have made an impact on the studies we captured. Additionally, the expanding application of health economic evaluations within the improvement and implementation sphere where high-quality study reporting has been a major recent focus has also plausibly contributed to improved reporting. Future evidence reviews will confirm whether this pattern is sustained over time.

### Strengths and limitations

This review offers an updated synthesis of an emerging field of economics evaluations of health care intervention evaluations covering both implementation and improvement science studies. The strict inclusion criteria mean that the reviewed evidence is cohesive. The systematic appraisal we carried out also allows us a longitudinal critique of the quality of economic studies in this field. Despite not being able to directly compare the quality assessment from the previous reviews, we would argue that the QHES used here is based on Drummond’s guidelines (used in prior reviews) and is designed to cover the same topics, but offers a simpler, quantifiable format that is easier to apply. [32]

This review has some limitations. First, while our search strategy was quite broad, our inclusion criteria were strict, which may have limited the number of studies that we identified and synthesised. We aimed to clearly demarcate the economic analyses carried out within healthcare implementation and improvement interventions research—and to explicitly include papers that included both costs and benefits, and so did not include cost-only studies. We also only considered papers reported in English. Taken together, these criteria are stricter than those applied to prior reviews, which were more inclusive of qualitative outcomes and costing studies.

### Implications for implementation and improvement research and future directions

Our review demonstrates an increasing number of health economic evaluations nested within implementation and improvement research studies, which further appear to be improving in methodological quality in recent years. Based on our review, we offer the following recommendations and areas for improvement in the continued application of health economic methods to improvement and implementation science evaluative research:

1. Utilise published guidance on conducting economic evaluation in implementation research and quality improvement projects. Existing implementation frameworks [40] make reference to the need to consider costs as part of an evaluative research strategy, but do not specify how this is to be done. The relationship between implementation outcomes, service outcomes and patient outcomes is central to understanding the benefits and costs and overall cost-effectiveness of an intervention.
2. Include detailed consideration of the measurement of the resource implications and ‘hidden’ costs relating to wider support activities required to initiate service improvement or to implement evidence into practice (e.g. costs of manualising an intervention; costs of developing and delivering train-the-trainers interventions as implementation strategies and so on).
3. Ensure that economic methods become fully integrated into the application of more recent methodological advancements in the evaluative design of improvement and implementation strategies, including ‘hybrid’ designs that seek to jointly test impact on implementation and patient outcomes. This would also provide an opportunity to explore the inter-linkages and relationships between implementation outcomes and economic measures of impact and the cost-effectiveness of improvement and implementation strategies.
4. While most of the economic studies included in this review were both ex-post and empirical, we would also highlight the value of ex-ante economic evaluation in policy-making contexts. This could be informative either at the early phase of an improvement or implementation project, to guide choices over which options are most likely to yield a cost-effective use of resources (and to rule out those that are likely to be excessively costly compared to expected benefits), or for quantifying the benefits and costs of spread of best practice and delivery at scale.
5. Finally, we would strongly recommend use of published guidelines and quality assurance frameworks to guide both the design and reporting of economic evaluations. Examples include the QHES framework (used here), the Consolidated Health Economic Reporting Standards (CHEERS) guidance [32] or the Drummond criteria [31].

## Conclusion

Economic evaluation can inform choices over whether and how resources should be allocated to improve services and for implementing evidence into health care practice. Our systematic review of the recent literature has shown that the quality of economic evidence in the field of improvement and implementation science has improved over time, though there remains scope for continued improvement in key areas and for increased collaboration between health economics and implementation science.

## Appendix

**Table 7:** Quality of health economic studies framework

Number	Question text	Scoring
1	Was the study objectively presented in a clear, specific and measurable manner?	Clear, specific, measurable = 7 Any two = 5 Any one = 2 None = 0
2	Was the perspective of the analysis (societal, third party, payer, etc.) and reasons for its selection stated?	Perspective = 2 Reasons = 2 Both = 4
3	Were variable estimates used in the analysis from the best available source (i.e. randomised control trial—best, expert opinion—worst)?	Randomised control trial = 8 Non-randomised control trial = 7 Cohort studies = 6 Case-control/case report/case series = 4 Expert opinion = 2
4	If estimates came from a subgroup analysis, were the groups prespecified at the beginning of the study?	Yes = 1 No = 0
5	Was uncertainty handled by (1) statistical analysis to address random events, (2) sensitivity analysis to cover a range of assumptions?	Statistical analysis = 4.5 Sensitivity analysis = 4.5 Both = 9
6	Was incremental analysis performed between alternatives for resources and costs?	Yes = 6 No = 0 CCA type of economic evaluation = NA
7	Was the methodology for data extraction (including the value of health states and other benefits) stated?	Yes = 5 No = 0
8	Did the analytic horizon allow time for all relevant and important outcomes? Were benefits and costs that went beyond 1 year discounted (3% to 5%) and justification given for the discount rate?	(1) Time horizon = 3 (2) Cost discounting = 1 (3) Benefit discounting = 1 (4) Justification = 2 All but justification = 5 All = 7
9	Was the measurement of costs appropriate and the methodology for the estimation of quantities and unit costs clearly described?	(1) Appropriateness of cost measurement = 4 (2) Clear description of methodology for the estimation of quantities = 2 (3) Clear description of methodology for the estimation of unit costs = 2 All = 8
10	Were the primary outcome measure(s) for the economic evaluation clearly stated and did they include the major short-term? Was justification given for the measures/scales used?	(1) Primary outcome clearly stated = 2 (2) Include major short-term outcome = 2 (3) Justification = 2 All = 6
11	Were the health outcomes measures/scales valid and reliable? If previously tested valid and reliable measures were not available, was justification given for the measures/scales used?	Yes = 7 No = 0
12	Were the economic model (including structure), study methods and analysis and the components of the numerator and denominator displayed in a clear, transparent manner?	(1) Economic model = 2 (2) Study methods = 1.5 (3) Analysis = 1.5 (4) Components of numerator = 1.5 (5) Components of denominator = 1.5 All = 8 If not a modelling study, done for (1) Study methods = 2 (2) Analysis = 2 (3) Components of numerator = 2 (4) Components of denominator = 2 All = 8
13	Were the choice of economic model, main assumptions and limitations of the study stated and justified?	(1) Economic model = 2 (2) Assumptions = 2.5 (3) Limitations = 2.5 All = 7 If not a modelling study, done (stated and justified) for (1) Assumptions = 3.5 (2) Limitations = 3.5 Both = 7
14	Did the author(s) explicitly discuss direction and magnitude of potential biases?	(1) Direction = 3 (2) Magnitude = 3 Both = 6
15	Were the conclusions/recommendations of the study justified and based on the study results?	Yes = 8 No = 0
16	Was there a statement disclosing the source of funding for the study?	Yes = 3 No = 0
